# A longitudinal study of work-related injuries: comparisons of health and work-related consequences between injured and uninjured aging United States adults

**DOI:** 10.1186/s40621-018-0166-7

**Published:** 2018-09-24

**Authors:** Navneet Kaur Baidwan, Susan G. Gerberich, Hyun Kim, Andrew D. Ryan, Timothy R. Church, Benjamin Capistrant

**Affiliations:** 1 Occupational Injury Prevention Research Program, Division of Environmental Health Sciences, School of Public Health, University of Minnesota, Minneapolis, MN USA; 2 Midwest Center for Occupational Health and Safety Education and Research Center, Division of Environmental Health Sciences, School of Public Health, University of Minnesota, Minneapolis, MN USA; 3 Division of Environmental Health Sciences, School of Public Health, University of Minnesota, Minneapolis, MN USA; 4 Epidemiology and Community Health, School of Public Health, University of Minnesota, Minneapolis, MN USA; 5 Statistical and Data Sciences, Smith College, Northampton, MA USA

**Keywords:** Physical work requirements, Occupational injuries, Functional limitations, Work status changes, Aging workers, Health retirement study

## Abstract

**Background:**

Age may affect one’s susceptibility to the myriad physical hazards that may pose risks for work-related injuries. Aging workers are not only at risk for work-related injuries but, also, at even higher risk for more severe health and work-related consequences. However, limited longitudinal research efforts have focused on such injuries among the aging workforce. This study aimed to investigate the association between physical work-related factors and injuries among United States (U.S.) workers, and then compare the injured and uninjured workers with regard to consequences including, functional limitations, and reduced working hours post injury. A cohort of 7212 U.S. workers aged 50 years and above from the U.S. Health and Retirement Study were retrospectively followed from 2004 to 2014. Data on exposures were lagged by one survey wave prior to the outcome of work-related injuries and consequences, respectively. Crude and adjusted incident rate ratios, and hazard ratios were estimated using generalized estimating equations and Cox models.

**Results:**

Risk of experiencing a work-related injury event was over two times greater among those whose job had work requirements for physical effort, lifting heavy loads, and stooping/kneeling/crouching, compared to those who did not. Over time, injured compared to uninjured workers had higher risks of functional limitations and working reduced hours.

**Conclusions:**

The aging workforce is at a high risk of experiencing injuries. Further, injured adults were not only more likely to incur a disability prohibiting daily life-related activities, over time, but, also, were more likely to work reduced hours. It will be important to consider accommodations to minimize functional limitations that may impair resulting productivity.

## Background

Work and hazards related to work may result in work-related injuries and compromise the health and safety of workers (Schulte et al., 2012). In the United States (U.S.) work-related injuries and illnesses, combined, have been estimated to cost $250 billion (Leigh, 2011). Several factors play an important role in affecting the overall health and safety of a worker, including age. Age, specifically, influences a worker’s susceptibility or resistance to various hazards to which they are exposed in the workplace (Schulte et al., 2012). With the overall U.S. population aging, the proportion of the aging working population is increasing and, by the year 2020, workers aged 55 years and above will comprise 25% of the workforce (Hayutin et al., 2013). Therefore, there is a need to address the potential risks for injuries among aging workers.

While workers aged 55 years and above experience more severe consequences as a result of injuries than their younger counterparts, the rates of non-fatal work-related injuries are lower among the older, compared to the younger group (Grandjean et al., 2006; Silverstein, 2008). As reported by the Bureau of Labor Statistics, work-related injuries resulted in over 1.1 million days-away-from-work cases in the year 2015 among the U.S. private industry and state and local governments. Importantly, workers aged 55–64 years, compared to all other age groups, had the highest incidence rate of days-away-from-work (115.8 cases per 10,000 full-time workers) (Bureau of Labor Statistics-Nonfatal Occupational Injuries and Illnesses Requiring Days Away From Work, 2016). In the same year, those aged 65 years and above had a fatal injury rate four-times that of workers in the age group of 25 to 34 years (Bureau of Labor Statistics-Census of Fatal Occupational Injuries, 2016).

An employee’s health and safety behaviors in the workplace are a result of interplay among various work requirements, including physical work requirements (Sorensen et al., 2011). Injuries are likely to occur in conditions where there is a mismatch between the capabilities of the employee and these work requirements (Silverstein, 2008) because requirements that do not match an employee’s abilities constrain an employee’s progress toward working safely (Hollander & Bell, 2010; Nahrgang et al., 2011). There is evidence that heavy physical work, lifting and forceful movements, bending and twisting, whole-body vibration, and static work postures are associated with back injuries. Further, repetition, force, and posture have been found to be associated with neck and neck/shoulder injuries (Bernard, 1997). Among the U.S. adults aged 50 years and older, about 44% have a job that requires physical effort almost all or most of the time, and another 25% are employed in a position that requires physical effort at least some of the time (Benz et al., 2013). Therefore, a large proportion of the aging U.S. workforce may be at a risk for injuries related to such physical work requirements.

Still, limited longitudinal research efforts have focused on physical work requirements and health and safety outcomes, including injuries, among the aging workforce. Since the majority of the existing research efforts have involved cross-sectional study designs, causal associations related to temporality cannot be made (Mann, 2003). Additionally, previous studies that investigated the association between physical work-requirement factors and injuries have been limited to certain specific occupational groups. For example, a study conducted among 31,076 material handlers, from 260 retail merchandise stores in the U.S., reported that workers in occupations with the greatest physical work requirements had an injury rate of 3.64 per 100 person-years versus 1.82 among workers with lesser requirements (Gardner et al., 1999).

Work-related injuries and illnesses may further lead to adverse personal life and work-related outcomes (Keogh et al., 2000; Dembe, 2001; Kim et al., 2017). However, there also remains a deficiency of quantitative literature assessing the consequences of such work-related injuries (Okechukwu et al., 2016). Existing research efforts have focused largely on workers’ compensation-related payments and return-to-work as the consequences of an injury. However, other less explored personal life-, health-, and work-related consequences of such injuries also need to be investigated (Keogh et al., 2000; Dembe, 2001). Many of the existing studies have compared health- and work-related consequences of injuries between aging and younger workers (Pransky et al., 2005; Algarni et al., 2015) but research efforts are still needed to compare such outcomes between injured and uninjured aging workers.

The aims of this study were, i) to analyze the potential associations between physical work-requirement factors and injuries, and ii) to explore the health-, and work-related consequences of such injuries among a cohort of U.S. workers aged 50 years and above while accounting for other socio-demographic, health-, and work-related characteristics that might influence these associations (Ghosh et al., 2004;, Baron et al., 2013; Kim et al. 2017).

## Methods

The data for this study were obtained from the Health and Retirement Study (HRS), a nationally-representative panel study of aging U.S. adults. The HRS which is a multistage area probability sample involves a representative sample of the U.S. population aged over 50 years and their spouses, has been surveying over 20,000 aging U.S. adults, since 1992, in biennial waves. Sampling weights have been provided to account for wave specific differential probability of selection and non-response (Sonnega et al., 2014).

### Study design

For the purpose of this study, HRS waves from the years 2004–2014 were used. Year 2004 was chosen as the starting point because, until 1998, two major HRS cohorts had not been combined, survey standardization did not commence until 2000, and the first sample replenishment year, since 1998, was 2004 (Sonnega et al., 2014). Year 2014 was chosen as the study end point because this was the last year for which complete data were available. Approval to conduct this study was obtained from the Institutional Review Board, University of Minnesota.

This research incorporates temporal causal assumptions (Hill, 1965) to examine the associations between the exposures and outcomes. Accordingly, to examine the association between work-requirement factors and injuries, injury data were obtained from waves subsequent to those from which the exposures were obtained. Thus, work-requirement factors were obtained from the years 2004–2012, and injury outcome data were obtained from 2006 to 2014. Similarly, data on any functional limitations and reduced working hours were obtained from waves subsequent to those from which injuries were obtained.

### Study sample

A total of 7212 adults, from a total of 20,000 HRS respondents aged 50 years and above, who participated in the HRS survey in the year 2004 and were working for pay in 2004, formed the cohort for this study. *For the first research question* investigating the association between physical work requirements and work-related injuries in the entire cohort, those who were not working for pay, at each survey wave, were excluded from the analyses. Also excluded were those who dropped out of the HRS sample (3.4%), and those who died (12%) over the study duration. *For the second research question* that investigated the association between work-related injuries and health-, and work-related outcomes, the entire original cohort of 7212 workers was retained, only dropping those who either died or dropped from the HRS study sample; those who stopped working for pay in the subsequent survey waves were retained. This was done to examine if being injured at any point in time during the study period would lead respondents to stop working for pay -- an important injury-related consequence.

### Study variables

All the study variables included in the analyses were self-reported. The primary exposures of interest for the first study aim were physical work-requirement factors, including work requirements for excessive physical effort, lifting heavy loads, and stooping kneeling crouching – all measured on a Likert scale, ranging from all/almost all of the time to none/almost none of the time. Missing information was imputed by carrying information from the last wave forward.

The outcome of interest for the first study aim was work-related injuries. These were ascertained as “(since the last interview wave have you had) any injuries at work that required special medical attention or treatment or interfered with your work activities?” Those who experienced a work-related injury were further asked about the number of such events. The current analyses uses injuries both as a binary outcome (yes/no), and as the number of such events (counts).

For the second study aim, injury status (injured versus uninjured) was the exposure of interest. The outcomes of interest were, i) any new functional limitations, ii) and reduced working hours. Functional limitations were assessed as having difficulties with five summary measures including, activities of daily living (bathing, eating, dressing, walking across a room, and getting in or out of bed); large muscle activity (sitting for two hours, getting up from a chair, stooping or kneeling or crouching, and pushing or pulling a large object); gross motor movements (walking one block, walking across the room, climbing one flight of stairs, and bathing); fine motor movements (picking up a dime, eating, and dressing); and mobility index (walking several blocks, walking one block, walking across the room, climbing several flights of stairs and climbing one flight of stairs). While HRS collected the counts of functional limitations, for this analysis, due to low cell counts these were categorized as a binary variable i.e., having any new functional limitation or not.

Reduced working hours was identified as a change to working fewer hours than in the previous interview wave. This also included those who partially or completely retired, as well as those who worked part-time in the following interview wave. As an example, those who changed work status from originally working full-time to part-time, or retiring in the subsequent wave, or from working part-time to retiring, were recognized as having reduced working hours.

Other potential confounding variables considered, for the analyses, included: demographic and health-related characteristics i.e., respondents’ age as of the survey wave, gender, race, ethnicity, education, and marital/partner status, and health-related information regarding presence of chronic physical and mental health conditions, and acute depression; and lifestyle factors of number of alcoholic drinks consumed per week, and smoking patterns; total household assets and income. Also included were other work-related characteristics, including: work category grouped as white collar, blue collar, and service; total hours worked during each wave; work status assessed as full-time, part-time, and partly-retired; having a second job; tenure in the current workplace; and history of any previous work-related injuries which could be predictors for future injuries. Further information on the measurement of each of these variables is presented in the later sections.

### Statistical analyses

**Fig. 1:**
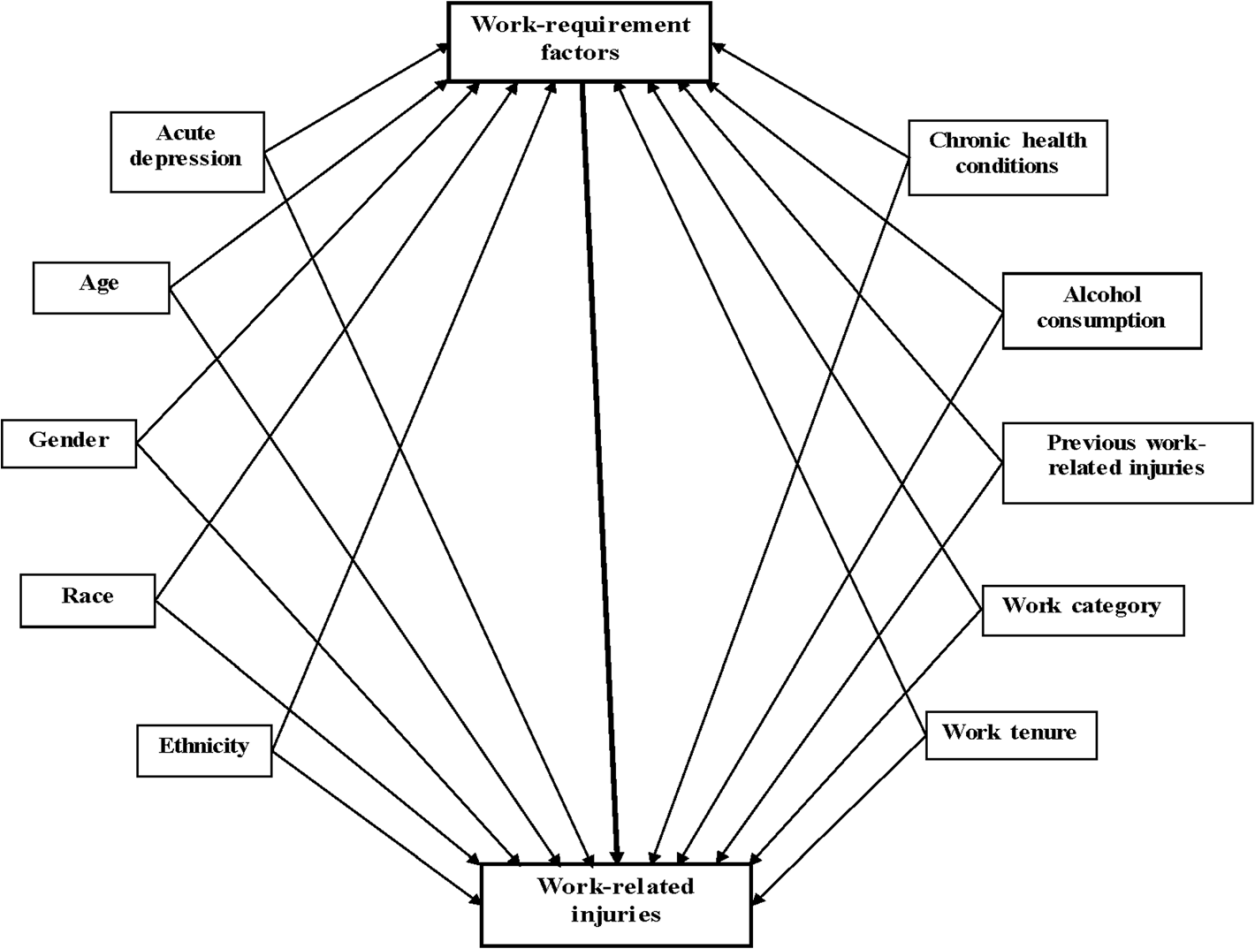
Directed acyclic graph representing work-requirement factors as the exposure and injuries as the outcome, along with confounding variables

Multivariable models were developed using Directed Acyclic Graphs (DAGs) that enable graphical displays of the a priori hypothesized causal links between the exposures of interest and the outcome. The DAGs helped to identify an essential set of confounding variables to adjust for in order to estimate the potential causal association between the exposure of interest and the outcome (Greenland et al., 1999). DAGs have previously been used for injury-related research, as well (Gerberich et al., 2001, Gerberich et al., 2014). Figure 1 represents a DAG example with work-requirement factors as the exposure of interest, and work-related injuries as the outcome, along with the set of essential confounding variables that must be considered in the analyses.

Work-related injuries were modeled both as the number of injury events (counts) and occurrence of injury (yes/no); respectively, incidence rate ratios (IRRs) and hazard ratios (HRs) were estimated. For estimating the IRRs, generalized estimating equations (GEEs) (Ballinger, 2004), with a negative binomial distribution of the errors and accounting for within-person and within-household correlations were used. HRs were obtained using Cox hazard models (Cox, 1972) with the counting process technique (Andersen & Gill, 1982), and accounting for within-person correlations. Changes, from the previous survey wave, in functional limitations and reduced working hours, were modeled as binary variables in terms of presence of any new functional limitation and reduced working hours. Risk ratios (RRs), instead of odds ratios (ORs) obtained from a log-binomial model, were used to model this association. This is because ORs are difficult to interpret and are non-collapsible. As an alternative, RRs are collapsible (i.e., without any confounders, a weighted average of stratum-specific ratios will be equal to the ratio obtained from a two-by- two table of pooled counts from stratum-specific tables), and easy to interpret (Cummings, 2009; Richardson et al., 2017). While sampling weights were obtained from the HRS, these were not used in the final analyses as these did not alter the study results. Note that, sensitivity analyses were conducted and the exposures of those who were censored were compared to those who were retained in the HRS survey. Additional sensitivity analyses compared the primary respondents with proxy respondents.

All analyses were conducted, using SAS statistical software (SAS, 2012).

## Results

At baseline, in 2004, about 5% (*n* = 397 of 7212 total) of the aging adults in this cohort, experienced a work-related injury. Most injured persons (63%) were in the age-group of 50–60 years, were White (77%) and Non-Hispanic (89%) (Table 1). Two-thirds of the injured persons had at least one or more chronic health conditions, and 58% had acute depression at the time of the survey. Table 1 also shows that the most common work categories, in which injured persons were engaged, included machine operators, transportation operators, and professional and technical services; 75% held full-time employment.

**Table 1:** Baseline demographic, other personal, and work-related characteristics among the uninjured and injured sample at the baseline (*N* = 7212)

Exposures	Uninjured *n* (%)	Injured *n* (%)
Age categories		
50–60 year old	3892 (56.9)	226 (63.3)
60–70 year old	2255 (33.0)	107 (30.0)
70 years and above	612 (9.0)	21 (5.9)
Gender		
Men	3375 (49.3)	168 (47.1)
Women	3465 (50.7)	189 (52.9)
Race		
White/Caucasian	5490 (80.3)	275 (77.0)
Black/African American	945 (13.8)	54 (15.1)
Other	403 (5.9)	28 (7.8)
Ethnicity		
Hispanic	594 (8.7)	38 (10.6)
Non-Hispanic	6245 (91.3)	319 (89.4)
Birthplace		
US born	6097 (89.1)	322 (90.2)
Born elsewhere	722 (10.6)	34 (9.5)
Education		
Left high-school/GED	1166 (17.0)	77 (21.6)
High-school graduate	1954 (28.6)	115 (32.2)
Some college	1698 (24.8)	95 (26.6)
College and above	2020 (29.5)	70 (19.6)
Marital status		
Married/partnered	5165 (75.5)	245 (68.6)
Separated/divorced/ widowed	1439 (21.0)	98 (27.4)
Never married	232 (3.4)	14 (3.9)
Total household assets ($)		
< =63,500	3731 (54.6)	239 (67.0)
> 63,500	3109 (45.5)	118 (33.1)
Alcohol consumption (drinks/week)		
None	4031 (58.9)	226 (63.3)
1–5	2715 (39.7)	122 (34.2)
6 or more	79 (1.2)	6 (1.7)
Chronic physical health conditions		
0	2216 (32.4)	90 (25.2)
1	2305 (33.7)	124 (34.7)
2 or more	2319 (34.0)	143 (40.1)
Acute depression		
No	3437 (50.2)	134 (37.5)
Yes	3117 (45.6)	207 (58.0)
Work category		
Managerial	1016 (14.8)	38 (10.6)
Professional/technical	1314 (19.2)	52 (14.6)
Sales	718 (10.5)	27 (7.6)
Clerical/administrative	1105 (16.1)	40 (11.2)
Health care	174 (2.5)	27 (7.6)
Protection service	121 (1.8)	11 (3.1)
Household/building cleaning service & Food preparation service	271 (4.0)	16 (4.5)
Personal service	438 (6.4)	26 (7.3)
Mechanical/Repair	202 (2.9)	12 (3.4)
Farming/forestry/fishing	200 (2.9)	18 (5.0)
Construction/Extraction	222 (3.2)	20 (5.6)
Precision production	184 (2.7)	9 (2.5)
Operators: machine, transportation	815 (11.9)	57 (16.0)
Work status		
Full-time	4391 (64.2)	270 (75.6)
Part-time	966 (14.1)	45 (12.6)
Partly retired	1483 (21.7)	42 (11.8)
Work tenure		
Five years or less	2966 (43.4)	128 (35.8)
More than five years	3486 (56.2)	229 (64.1)
Work-requirement factors: Does your job require		
*Excessive physical effort?*		
All/almost all the time	1136 (16.6)	98 (27.4)
Most of the time	822 (12.0)	64 (17.9)
Some of the time	1799 (26.3)	95 (26.6)
None/almost none of the time	2255 (33.0)	64 (17.9)
*Lifting heavy loads?*		
All/almost all the time	495 (7.2)	54 (15.1)
Most of the time	349 (5.1)	27 (7.6)
Some of the time	1418 (20.7)	107 (30.0)
None/almost none of the time	3750 (54.8)	133 (37.2)
*Stooping/kneeling/crouching?*		
All/almost all the time	916 (13.4)	94 (26.3)
Most of the time	609 (9.0)	47 (13.2)
Some of the time	1972 (28.8)	101 (28.3)
None/almost none of the time	2516 (36.8)	79 (22.1)
Total	6840 (94.8)	357 (4.9)

Missing values are not shown

Table 2 presents the results from the crude and adjusted GEE and Cox models, modeling the associations between physical work-requirement factors i.e., work requirements for excessive physical effort, lifting heavy loads, and stooping/kneeling/crouching, and the outcome of injuries. Compared with those whose workplaces did not include the three work requirements, those who had these requirements had a significantly higher risk of experiencing injuries (Table 2). Results of both the GEE and Cox models show that as the work requirements increased from “some of the time” to “all or almost all of the time,” the risk of injuries increased, as well.

**Table 2:** Analysis of the association between physical work-requirement factors and work-related injuries (N = 7212)

	^a^Outcome: Number of injury events		^b^Outcome: Injured or not	
	Univariate IRRs	Multivariable IRRs	Univariate HRs	Multivariable HRs
WORK-REQUIREMENT FACTORS: Does your work require				
*Excessive physical effort?*				
All/almost all the time	3.96 (3.15, 4.97)	2.19 (1.57, 3.05)	3.42 (2.80, 4.18)	2.32 (1.77, 3.03)
Most of the time	2.91 (2.25, 3.74)	1.71 (1.19, 2.46)	2.48 (1.99, 3.08)	1.84 (1.37, 2.47)
Some of the time	1.83 (1.47, 2.28)	1.46 (1.11, 1.91)	1.77 (1.45, 2.15)	1.59 (1.24, 2.02)
None/almost none of the time	1	1	1	1
*Lifting heavy loads?*				
All/almost all the time	3.88 (3.15, 4.45)	2.27 (1.60, 3.24)	3.35 (2.75, 4.09)	2.52 (1.88, 3.39)
Most of the time	2.12 (1.62, 2.77)	1.69 (1.14, 2.49)	2.24 (1.74, 2.89)	1.81 (1.27, 2.58)
Some of the time	2.26 (1.88, 2.70)	1.74 (1.37, 2.21)	2.12 (1.81, 2.47)	1.89 (1.54, 2.31)
None/almost none of the time	1	1	1	1
*Stooping/kneeling/crouching?*				
All/almost all the time	3.88 (3.16, 4.78)	2.20 (1.61, 3.01)	3.30 (2.72, 3.99)	2.41 (1.83, 3.15)
Most of the time	2.77 (2.10, 3.65)	1.87 (1.33, 2.61)	2.51 (2.01, 3.12)	2.01 (1.58, 2.79)
Some of the time	1.80 (1.49, 2.19)	1.46 (1.13, 1.89)	1.90 (1.59, 2.28)	1.67 (1.33, 2.09)
None/almost none of the time	1	1	1	1

^a^GEE models with negative binomial distribution: adjusted for age; gender; race; ethnicity; chronic physical and mental health conditions; acute depression; alcohol consumption; work category; work tenure; and previous history of work-related injuries (hours worked was the offset or exposure time)

^b^Cox models: age was used as the time to follow-up variable; other variables adjusted for were same as the GEE models

Table 3 shows results from the GEE models, comparing injured and uninjured aging adults in the study in terms of any new functional limitations incurred, and reduced working hours. In general, adjusted models showed that injured, compared with uninjured, aging workers were more likely to experience new functional limitations, and to work reduced hours. For example, injured, compared with uninjured persons, were almost twice as likely to have a difficulty with activities of daily living. Note that due to model convergence issues, a parsimonious set of confounding variables selected using the DAG shown earlier were included in this part of the analysis (Table 3).

**Table 3:** Comparing functional limitations and working hours among the injured and uninjured persons (*N* = 7212)

	^a^Comparing injured and uninjured aging workers for any new functional limitations and reduced working hours	
	Univariate RRs	Multivariable RRs
^b^Functional limitations - Presence of any difficulty with		
Activities of daily living		
Injured vs Uninjured	1.75 (1.42, 2.15)	1.78 (1.44, 2.19)
Large muscle index		
Injured vs Uninjured	1.20 (1.06, 1.36)	1.16 (1.01, 1.32)
Gross motor skills		
Injured vs Uninjured	1.57 (1.33, 1.86)	1.57 (1.33, 1.85)
Fine motor skills		
Injured vs Uninjured	1.86 (1.52, 2.27)	1.94 (1.58, 2.37)
Mobility index		
Injured vs Uninjured	1.31 (1.15, 1.48)	1.32 (1.16, 1.50)
^c^Reduced working hours		
Injured vs Uninjured	0.97 (0.87, 1.07)	1.19 (1.10, 1.30)

^a^GEE models with log-binomial distribution

^b^Adjusted for age, gender, race, education, chronic physical and mental health conditions, work category, and hours worked

^c^Additionally adjusted for having a second job

## Discussion

Results of the longitudinal cohort study analyses indicated that the risk of work-related injuries among the aging workers increased as the work requirements for excessive physical effort, lifting heavy loads, and stooping/kneeling/crouching increased. Specifically, the risk of injuries among those whose work had these physical work requirements “all or almost all the time,” was two-times that of those whose work did not have such requirements.

Similarly, from an earlier analysis of a cohort of 51–61 year old non-farmers in the HRS dataset whose work required heavy lifting, compared to those whose work did not, a risk of having a work-related injury was over two times greater (Zwerling et al., 1996, Zwerling et al., 1998). A cross-sectional study conducted, using data from the U.S. National Longitudinal Survey of Youth (NLSY), also found that those whose workplaces encompassed the stated physical work requirements were also about twice as likely to experience injuries at their workplaces (Dembe et al., 2004); this compares to a three-fold high risk observed in the current study.

A study conducted among six industrial sectors that were part of the Israeli Cardiovascular Occupational Risk Factors Determination in Israel, reported that the incidence of injuries increased with increasing levels of work-related physical stress involved (Melamed et al., 1999). Another study (Hollander & Bell, 2010), that specifically focused on the U.S. Army, documented that soldiers in heavy versus light demanding work were at a higher risk for any cause of injuries and disabilities (HR: 1.45, 95% CI: 1.34, 1.57).

As noted earlier, work-related injuries and illnesses can be associated with several health and work-related consequences, including functional impairments, disabilities, job loss, absenteeism etc. (Dembe, 2001, Keogh et al., 2000). However, the majority of previous research relied on Workers’ Compensation data to investigate such outcomes. Therefore, injured and uninjured populations could not be compared in terms of any functional limitations, or work hour changes. For example, a previous study, focused on Workers’ Compensation claims and investigated the consequences of upper extremity cumulative trauma disorders (Keogh et al., 2000); it was found that one to four years following claims filing, more than half of the claimants reported having symptoms that interfered with work (50%+) and recreational (60%+) activities. Further, only 64% reported being able to perform normal activities of daily living. Results also showed that the likelihood of normal function decreased with increasing age (OR: 0.94, CI: 0.91, 0.97). In addition, approximately 40% reported job loss one to four years post-claims filing.

However, the current research compared such consequences between aging injured and uninjured employees, and found that injured employees had a higher risk of experiencing functional limitations, and reduced working hours than the previous survey wave. Similar results were documented from another study that used data from the Work, Family and Health Network, and investigated the association between occupational injuries and job loss (Okechukwu et al., 2016). It was reported the risk of having an involuntary job loss, as a consequence of the injury, was twice as high among the injured, compared to the uninjured, workers (OR: 2.19; CI: 1.27, 3.77). Similar results were also obtained from a study that sampled newly registered hospital nurses in the U.S. and found that those experiencing work-related sprains and strains, including back injuries, were more likely to report subsequent job loss (Brewer et al., 2012). Contrary to these findings, a study that focused solely on male workers, using the U.S. NLSY, found no association between injuries and job loss among unionized workers (Woock, 2009).

This study has several strengths owing to its focus on the aging U.S. workforce, and use of longitudinal analysis techniques. However the findings from this study must be interpreted in view of some of the limitations. First, the data are based on self-reports and also involve a minimum of two-year recall periods. Therefore, there may be a potential for differential misclassification. This is because the estimates may be biased away from the null among those who experienced a work-related injury-related event as they may remember their exposures better than those who did not experience such injuries. It is also possible that those who were censored over the study period may be different from those who were retained in terms of their exposures. However, sensitivity analyses revealed that injured/uninjured and censored/non-censored were similar in terms of their exposures. It is also possible that there could be some bias in the estimates associated with proxy interviews. While the results of this study would be considered generalizable to the U.S., it cannot be compared to other country data. The results would also not be expected to be generalizable to younger working populations, or other work groups due to potentially different exposures.

## Conclusions

This unique longitudinal research effort serves as a basis to provide insights into work-related injury experiences and their consequences among aging U.S. workers, whose proportion in the workforce is increasing. The risk of work-related injuries is especially high among aging U.S. workers employed in physically demanding jobs. The aging workforce is likely to be very experienced, knowledgeable and skilled (Eyster et al., 2008). Employers therefore, must consider providing accommodations for workers, relevant to work requirements, to promote optimal efficiency and prevent functional limitations that may impair resulting productivity (Eyster et al., 2008, Dong, 2018). Research suggests providing flexible work arrangements using strategies such as: part-time work; flexibility to change jobs within the company; job sharing; and telework (Eyster et al., 2008).
